# Association of Increase in White Matter Hyperintensity Volume With Rate of Hippocampal Atrophy in a Population-Based Study of Aging

**DOI:** 10.1212/WNL.0000000000213975

**Published:** 2025-08-19

**Authors:** Thomas M. Brown, Sarah-Naomi James, Jennifer M. Nicholas, Sarah Keuss, Ashvini Keshavan, Ian B. Malone, William Coath, Heidi Murray-Smith, David M. Cash, Frederik Barkhof, Marcus Richards, Jonathan M. Schott, Carole H. Sudre, Josephine Barnes

**Affiliations:** 1 Dementia Research Centre, UCL Queen Square Institute of Neurology, London, United Kingdom;; 2 Unit for Lifelong Health and Ageing at UCL, Department of Population Science and Experimental Medicine, University College London, United Kingdom;; 3 Department of Medical Statistics, London School of Hygiene and Tropical Medicine, United Kingdom;; 4 UK Dementia Research Institute at University College London, United Kingdom;; 5 Hawkes Institute, Department of Medical Physics and Biomedical Engineering, University College London, United Kingdom;; 6 Department of Radiology & Nuclear Medicine, Amsterdam UMC, Vrije Universiteit, the Netherlands; and; 7 School of Biomedical Engineering and Imaging Sciences, King's College London, United Kingdom.

## Abstract

**Background and Objectives:**

Higher white matter hyperintensity volume (WMHV) is associated with hippocampal atrophy, cognitive decline, and dementia; however, it is unknown whether continually increasing WMHV is related to hippocampal atrophy. The aim of this study was to determine whether higher WMHV change rate (WMHVR) is related to higher hippocampal atrophy rate (HAR); this relationship is dependent on cardiovascular risk factors (CVRFs), Alzheimer disease (AD) pathology, and genetic risk; and this relationship is mediated by neuroaxonal degradation.

**Methods:**

Participants from Insight46, a substudy of the 1946 British Birth Cohort, underwent combined [^18^F]florbetapir PET/MRI scans at University College London approximately 2.5 years apart. WMHVR was quantified from T2/fluid-attenuated inversion recovery and HAR from T1 sequences. Life-course blood pressure and CVRF data were measured at 6 and 3 time points, respectively. *APOE* genotype and neurofilament light chain (NfL) quantification were derived from blood samples. Participants with neurologic conditions were excluded from primary analyses. Linear regression was used to test the relationships between WMHVR and HAR, adjusting for sex, age, and total intracranial volume (TIV) and CVRF, *APOE*-ε4 status, and β-amyloid (Aβ) in separate models. Semipartial *R*^2^ was calculated from these models. In a post hoc analysis, structural equation modeling aimed to determine whether NfL mediated the relationship between WMHVR and HAR.

**Results:**

A total of 317 individuals without neurologic conditions (48% female, 100% White British, mean baseline age [SD] = 70.5 [0.6] years) were included. The mean HAR was 0.04 [0.04] mL/y. Each 1 mL/y increase in WMHVR was associated with a 0.014 mL/y (95% CI 0.005–0.022) increase in HAR, adjusted for TIV, age, and sex (*p* = 0.002). Adjustment for additional variables did not meaningfully attenuate this association (≥0.012 mL/y, *p* ≤ 0.005, all models), and there was no indirect effect through NfL (0.0004 mL/y [95% CI −0.0006 to 0.0012], *p* < 0.1).

**Discussion:**

Higher WMHVR was associated with HAR between approximately 70 and 73 years, independent of CVRF levels, *APOE*-ε4 status, and Aβ load, and not mediated by markers of neuroaxonal degradation. Although AD-specific pathology is typically considered the main cause of accelerated HAR, we demonstrated that HAR is also linked to deteriorating WM health. These results will need to be replicated in more diverse cohorts with longer follow-up periods to confirm the findings.

## Introduction

Fifty-five million people are living with dementia globally,^1^ with an estimated 60%–70% of these cases caused by Alzheimer disease (AD). Using neuroimaging biomarkers that can identify relevant brain changes before advanced symptom onset will aid diagnosis when interventions are most likely to be effective. Within the A/T/N biomarker framework,^2^ β-amyloid (Aβ) and hyperphosphorylated tau (p-tau) are considered AD-specific drivers of neurodegeneration and cognitive impairment. In addition, non–AD-specific processes including cerebral small vessel disease (cSVD) have been shown to further accelerate progression toward AD.^3,4^

White matter hyperintensities (WMHs) are areas of hyperintense signal on T2-weighted fluid-attenuated inversion recovery (FLAIR) MRI. They are common findings in older patients; although they have many potential causes such as autoimmune demyelination, Wallerian degeneration, or ependymal denudation, WMHs of presumed vascular origin are primarily caused by cSVD. In these cases, arteriosclerosis and ischemia can lead to hypoperfusion, hypoxia, altered cerebral autoregulation, increased blood-brain barrier permeability, and inflammation that damage WM fibers, leading ultimately to demyelination and WMHs.^5^

Cardiovascular risk factors (CVRFs) including age, hypertension, obesity, smoking, diabetes, hypercholesterolemia, and sedentary lifestyles are associated with higher WMH volume (WMHV).^6^ Blood pressure (BP) is particularly strongly linked to higher WMHV, with higher BP across adulthood predictive of later life WMHV.^7^ Many of these risk factors have also been highlighted as potentially modifiable risk factors of dementia.^8^

Higher WMHV is associated with cognitive impairment, AD, and all-cause dementia.^4^ There is mixed evidence regarding whether WMHs and Aβ accumulation are directly related, synergistic processes, or whether they have independent, additive effects on cognition and AD development.^9,10^ Higher WMHV is also associated with increased brain atrophy, particularly in the hippocampus,^11,12^ one of the earliest affected brain regions in sporadic AD.^13^ Hippocampal atrophy is associated with cognitive impairment and correlates with Braak staging,^14^ *APOE*-ε4 status, and levels of CSF Aβ.^15^

Previous research in Insight46 has shown that higher WMHV accumulated by approximately age 70 is related to higher HAR.^11^ However, it is not known whether continually increasing WMHV beyond age 70 is also related to increased HAR. Investigating these relationships will determine the consequences of continually deteriorating WM health and whether tracking these changes is important for monitoring AD risk in presymptomatic individuals. In this study, we investigated whether a higher WMHV change rate (WMHVR) was associated with a greater hippocampal atrophy rate (HAR) between approximately 70 and 73 years and tested whether this relationship is dependent on levels of life-course CVRF (which are known to influence WMHV), AD pathology, and genetic risk (which are known to influence HAR). In addition, the mechanisms underlying relationships between WMHV and gray matter atrophy are not well understood, and it is not known whether these 2 variables are linked by WMHs, reflecting neuroaxonal degradation, resulting in a reduction in hippocampal connections and subsequently atrophy. In a post hoc analysis, we aimed to determine whether the hypothesized relationship between WMHVR and HAR was mediated by neuroaxonal degradation, measured using plasma neurofilament light chain (NfL).

We hypothesized that, in this cohort of older adults without neurologic disorders, higher WMHVR would be related to increased HAR, and that this relationship would be partly dependent on levels of mid-life CVRF but independent of Aβ levels and *APOE*-ε4 status. The secondary hypothesis was that axonal degradation may be the mechanistic link between WMHVR and HAR (whereby WMHVR reflects axonal damage and damage to WM fibers results in poorer connectivity to the hippocampus, causing it to atrophy at a faster rate).

## Methods

### Data Collection

Participants in this study were recruited from the National Survey of Health and Development (NSHD) 1946 British Birth Cohort.^16^ All NSHD participants were born in Great Britain in the same week of March 1946 and have undergone regular data collection throughout life. Since 2015, a neuroscience substudy of the NSHD, Insight46, has been conducted at University College London (UCL), focused on identifying risk factors affecting brain health, cognition, and AD risk. Two phases of data collection are complete for Insight 46, and a third phase is ongoing. Full details of the study protocol and recruitment procedure are available elsewhere^17-19^ and summarized further.

#### Phase 1

A total of 502 participants who remained within the NSHD at age 69 years and who had consistent data collection throughout life took part in a research clinic visit at UCL between May 2015 and January 2018. Participants underwent detailed neuropsychological assessment, neurologic examination, and a combined PET/MRI brain scan.

#### Phase 2

Of the 502 participants who took part in phase 1 and were invited for follow-up assessments, 442 returned for repeat testing between January 2018 and January 2021. Twenty-nine of these participants were assessed remotely because of the coronavirus disease 2019 pandemic and, therefore, did not have PET/MRI scans.

### Standard Protocol Approvals, Registrations, and Patient Consents

Ethical approval for the study was granted by the Health Research Authority Research Ethics Committee London (REC reference 14/LO/1173), and all participants provided written informed consent.^17,18^

### Neuroimaging Procedures

Scans at both time points were performed on a Biograph mMR 3T PET/MRI scanner (Siemens Healthcare), with PET and MRI data obtained simultaneously over a single 1-hour scan. Participants were intravenously injected with 370 MBq of the Aβ-binding PET tracer [^18^F]florbetapir (Amyvid). PET data were acquired continuously for the full duration of the scan.

T1-weighted and T2-FLAIR volumetric MRI sequences underwent gradient nonlinearity, brain-masked N4-bias correction, and visual quality control.^17^ For each participant, a mid-point space across time points was created to which T1 and FLAIR images were co-registered, and an automated longitudinal WMH segmentation algorithm (Bayesian Model Selection [BaMoS]) was applied to generate WMH masks.^20^ These masks underwent pairwise visual quality control and were corrected automatically using modified scripts if segmentation issues were identified. WMHVs were extracted from these masks at both time points, and WMHVR was calculated by subtracting baseline WMHV from follow-up WMHV and dividing by scan interval (years).

Hippocampal atrophy was calculated using k-means–normalized (KN) boundary shift integral (BSI). BSI measures changes in normalized intensity at anatomical boundaries defined using segmentation of co-registered T1 MR images and then calculates the shift of these boundaries between the 2 time points to give a measurement of brain atrophy.^21^ The KN-BSI method was developed to improve the accuracy of classic BSI measurements by performing tissue-specific intensity normalization between the 2 co-registered images.^22^ To calculate hippocampal BSI, the hippocampi from each time point were further co-registered and hippocampal BSI was calculated using a double intensity window approach.^23^ The results were quantified in milliliters of hippocampal volume change, and the volume change of both hippocampi was summed to give a measurement of total hippocampal atrophy.

Aβ PET images (50–60 minutes after injection) were registered to the T1 image, and partial volume correction was applied using a T1 parcellation and the iterative Yang method.^24^ Standardized uptake value ratios (SUVRs) were calculated using a whole cerebellar reference region and a cortical composite target region and then transformed to Centiloid (CL) scores.^25^ Binary Aβ status at phase 1 was assigned using a bi-Gaussian mixture modeling–derived cutoff value of 11.9 CLs, representing the 99th percentile of the lower (negative) distribution.

### Cardiovascular Risk Factors

As part of the NSHD assessments, participants had seated systolic BP (SBP) and diastolic BP (DBP) measured by research nurses during home visits at ages 36, 43, 53, 60–64, and 69.^26^ In addition, lying and standing BP was measured at Insight46 phase 1.^17^ Framingham Heart Study Cardiovascular Risk Score (FHS-CVS)^27^—based on age, sex, SBP, antihypertensive medication, diabetic status, current smoking status, and body mass index—was calculated at ages 36, 53, and 69.

### Biological Samples

DNA was extracted from previously collected whole blood using a standard phenol-chloroform method, and *APOE* genotyping was performed.^28^ Participants were classified as either *APOE*-ε4 carriers or noncarriers.

EDTA plasma was obtained from nonfasted participants at Insight 46 phase 1^28^ and stored at −80 C within 60 minutes of sampling. Plasma concentrations of NfL, a nonspecific marker of neuroaxonal damage, were quantified in singlicate using commercially available multiplex assay kits (Neuro 4-Plex E, Quanterix) on a Simoa HD-X platform, according to the manufacturer's instructions. Further details are provided in eMethods 1 and eTable 1.

### Statistical Analyses

**Figure 1:**
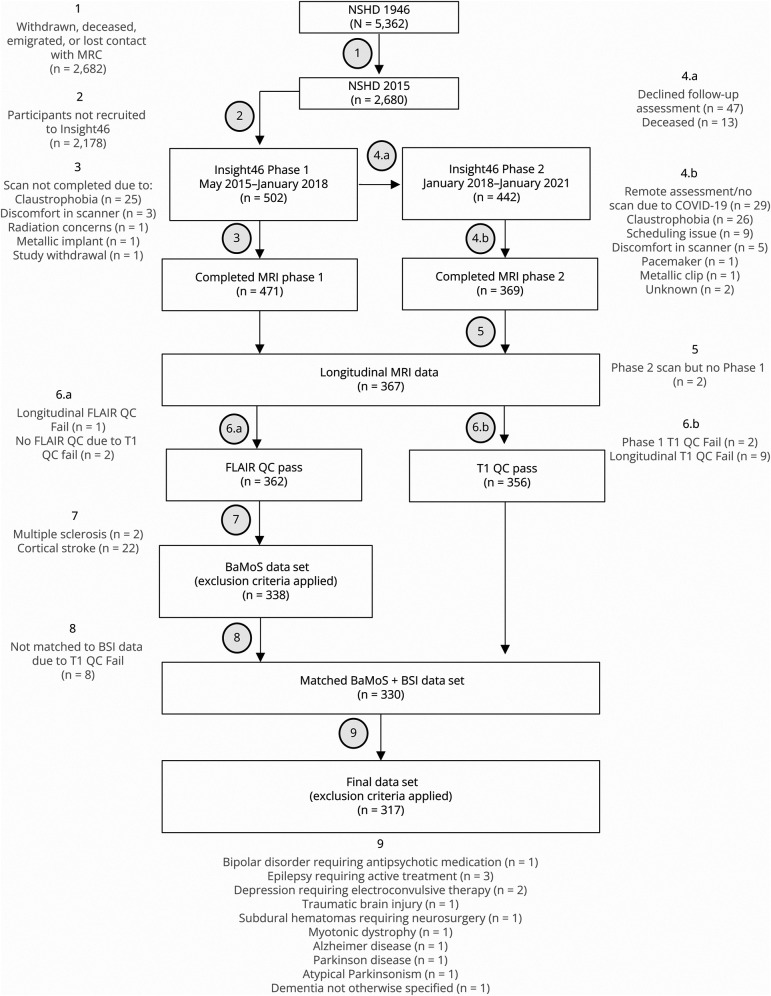
Flowchart Showing Participant Recruitment to Insight46, MRI QC, and Exclusion Criteria At stage 7, individuals with evidence of cortical stroke or autoimmune demyelination were excluded from the longitudinal WMH data set to prevent the inclusion of other imaging characteristics (stroke and MS lesions) mimicking WMH. At stage 9, individuals with evidence of any other neurologic condition were excluded to prevent these individuals, in whom WMH and hippocampal atrophy are likely to be higher, from driving associations. BSI = boundary shift integral; FLAIR = fluid-attenuated inversion recovery; MS = multiple sclerosis; QC = quality control; WMH = white matter hyperintensity.

Participants with stroke or multiple sclerosis (MS) lesions were excluded from the WMHV data to prevent these lesions being mischaracterized as cSVD-related WMHs. Individuals with other neurologic disorders were excluded from the main analysis to prevent these individuals, in whom WMHVR and HAR are likely to be higher, from driving associations in the results. A flowchart of participant recruitment and inclusion/exclusion criteria is presented in Figure 1. Individuals with neurologic disorders (other than stroke or MS) were included later in a sensitivity analysis.

Statistical analyses were conducted in Stata 18.0. Linear regression with Huber and White robust standard errors to account for heteroscedasticity was used for all models. First, we aimed to determine whether baseline (phase 1) WMHV (independent variable) was related to WMHVR (dependent variable), while adjusting for total intracranial volume (TIV), sex, and age at phase 1. Because WMHVR is non-normally distributed, the data were log-transformed when used as a dependent variable (in this model only).

Linear regression was then used to determine associations between WMHVR and HAR, adjusting for TIV, sex, age at phase 1, and scan interval. Hippocampal atrophy was the dependent variable, with scan interval included as a covariate to estimate HAR. WMHVR and all other independent variables were allowed to interact with the scan interval to model their influence on HAR. As the dependent variable was change between visits, no constant term was included because it is assumed that change over an interval of zero years would itself be zero (as described previously in other studies^11,29^). This model was repeated with the addition of other variables of interest (each interacted with scan interval) in separate models (Table 1) to examine whether any potential association between WMHVR and HAR was dependent on levels of these variables. A model adjusting for whole-brain BSI was included (Table 1, model 1.2) to determine whether WMHVR disproportionately affected HAR, beyond the effects of WMHVR on brain atrophy rate as a whole. After inspection of the linear regression outputs and plots, no influential data points were identified and all participants with available data were included in each analysis. Further details on this statistical approach are available in eMethods 2 and eFigures 1 and 2.

**Table 1:** Linear Regression Models to Test the Influence of WMHVR and Additional Variables of Interest on HAR as an Outcome

Model	Independent variables	N
1.1	WMHVR	317
1.2	WMHVR + WB BSI	317
2.1.1	WMHVR + SBP age 36 (seated)	286
2.1.2	WMHVR + DBP age 36 (seated)	286
2.2.1	WMHVR + SBP age 43 (seated)	303
2.2.2	WMHVR + DBP age 43 (seated)	303
2.3.1	WMHVR + SBP age 53 (seated)	310
2.3.2	WMHVR + DBP age 53 (seated)	310
2.4.1	WMHVR + SBP age 60–64 (seated)	317
2.4.2	WMHVR + DBP age 60–64 (seated)	317
2.5.1	WMHVR + SBP age 69 (seated)	315
2.5.2	WMHVR + DBP age 69 (seated)	315
2.6.1	WMHVR + SBP age 70–73 (lying)	315
2.6.2	WMHVR + DBP age 70–73 (lying)	315
2.7.1	WMHVR + SBP age 70–73 (standing)	315
2.7.2	WMHVR + DBP age 70–73 (standing)	315
3.1	WMHVR + FHS-CVS age 36	286
3.2	WMHVR + FHS-CVS age 53	309
3.3	WMHVR + FHS-CVS age 69	312
4	WMHVR + *APOE*-ε4 status	316
5	WMHVR + baseline (phase 1) Aβ CL score	311

Abbreviations: Aβ = β-amyloid; CL = Centiloid; DBP = diastolic blood pressure; FHS-CVS = Framingham Heart Study Cardiovascular Risk Score; HAR = hippocampal atrophy rate; NfL = neurofilament light chain; SBP = systolic blood pressure; TIV = total intracranial volume; WB BSI = whole-brain boundary shift integral; WMHV = white matter hyperintensity volume; WMHVR = WMHV change rate.

Covariates sex, age, and TIV were included in all models, and all models had HAR as the dependent variable. All independent variables and covariates were included as an interaction term with scan interval. Huber and White robust standard errors were used in all models. The same variables were further accounted for in semipartial *R*^2^ analysis, again in separate models.

A fully adjusted linear regression model was then run with WMHVR and all additional variables and covariates in Table 1, models 2–5 as predictors in the same model to determine the combined effects of all variables on the relationship between WMHVR and HAR. The individual effects of BP and FHS-CVS measurements in this model cannot be individually interpreted in the same way as the separate models because of multicollinearity. Regression model 5 was repeated but with the inclusion of a three-way interaction between WMHVR, Aβ CL score, and scan interval. The main linear regression analyses (Table 1) were repeated in a complete case subset containing only individuals who had all observations from all measurements, to check that the differences in WMHVR coefficients when accounting for additional variables of interest were not due to only sample size changes. The complete case subset was not used for the primary analysis because of the reduction in sample size.

A sensitivity analysis was conducted that included individuals with neurologic disorders other than stroke or MS. Additional participants included in the sensitivity analysis had one of the following neurologic conditions: bipolar disorder requiring antipsychotic medication (N = 1); epilepsy requiring active treatment (N = 3); depression requiring electroconvulsive therapy (N = 2); traumatic brain injury (N = 1); subdural hematoma requiring neurosurgery (N = 1); myotonic dystrophy (N = 1); AD (N = 1); Parkinson disease (N = 1); atypical Parkinsonism (N = 1); dementia not otherwise specified (N = 1). Individuals with stroke or MS were excluded from the WMHV data set at a previous stage because the WMHV measures are strongly influenced by these conditions and so were not included in this sensitivity analysis.

In a separate analysis, *R*^2^ and semipartial *R*^2^ were derived from the linear regression models to determine the percentage of variance in HAR explained by all independent variables combined (*R*^2^) and the percentage of variance in HAR uniquely explained by WMHVR when further adjusting for each other variable (Table 1) in separate models as well as all additional variables in a fully adjusted model.

In a post hoc analysis, we aimed to determine whether neuroaxonal damage (measured using plasma NfL at follow-up [phase 2]) mediated the hypothesized relationship between WMHVR and HAR. We used a structural equation model to determine the indirect effects of WMHVR on HAR with phase 2 NfL (eFigure 3), using robust standard errors and no constant term, similar to the linear regression models. Because NfL was non-normally distributed, it was log_2_ transformed before being used in the model (so that the effect on HAR would be expressed as that associated with each doubling of NfL in plasma). In this model, hippocampal atrophy was divided by scan interval (years) to reflect the rate of hippocampal atrophy per year.

### Data availability

Anonymized data are available on request.^30^ 

## Results

### Participants and Sample Characteristics

**Figure 2:**
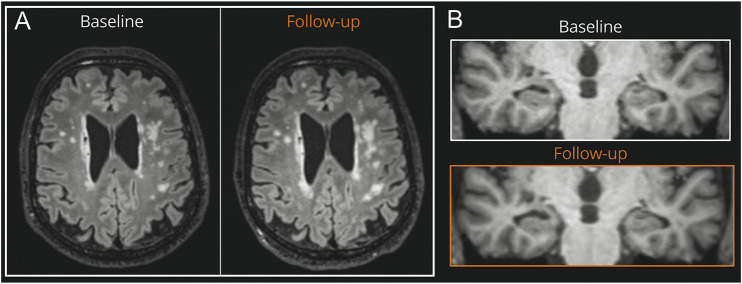
Example of WMHV Change and Hippocampal Atrophy An example of widespread WMH growth on co-registered axial FLAIR images (A) and hippocampal atrophy on coronal T1 images of the temporal lobes (B) at baseline and follow-up 2.5 years later. In this individual, the WMHVR was 4.6 mL/y, the baseline total hippocampal volume was 6.6 mL, and the HAR was 0.17 mL/y. FLAIR = fluid-attenuated inversion recovery; WMH = white matter hyperintensity; WMHV = WMH volume; WMHVR = WMHV change rate.

A total of 317 individuals (mean [SD] age at baseline 70.5 [0.6] years; 48% female; 100% White British) with high-resolution isotropic MRI data at 2 time points (mean [SD] scan interval 2.4 [0.2] years) and without the diagnosis of a neurologic disorder were analyzed in this study (Table 2). Examples of WMHV changes and hippocampal atrophy are shown in Figure 2. Distributions of WMHV and HAR data are shown in eFigure 4.

**Table 2:** Summary of Demographics, Biomarkers, and Imaging Outcomes

	N	Frequency (%)	Mean (SD)	Median (IQR)
Ethnicity, frequency (%) White British	317	317 (100)	—	—
Sex, frequency (%) female	317	153 (48)	—	—
Age at baseline (phase 1) scan, y	317	—	70.5 (0.6)	70.5 (70.0 to 71.0)
Scan interval, y	317	—	2.4 (0.2)	2.4 (2.3 to 2.5)
Baseline (phase 1) WMHV, mL	317	—	5.7 (5.9)	3.51 (1.81 to 7.50)
WMHV change rate, mL/y	317	—	1.0 (1.6)	0.22 (0.05 to 0.56)
Hippocampal atrophy rate, mL/y	317	—	0.04 (0.04)	0.04 (0.01 to 0.06)
Baseline (phase 1) Aβ status, frequency (%) positive	311	74 (24)	—	—
Baseline (phase 1) Aβ CL score	311	—	(9.6–22.7)	0.9 (−3.4 to 10.4)
*APOE-*ε4 status, frequency (%) ε4 carrier	316	95 (30)	—	—
Systolic BP, mm Hg				
Age 36, seated	286	—	120 (14)	119 (110 to 130)
Age 43, seated	303	—	123 (14)	123 (114 to 130)
Age 53, seated	310	—	133 (19)	131 (120 to 148)
Age 60–64, seated	317	—	134 (17)	133 (123 to 145)
Age 69, seated	315	—	132 (16)	132 (122 to 143)
Age 70, lying	315	—	137 (17)	136 (126 to 147)
Age 70, standing	315	—	137 (19)	135 (123 to 149)
Diastolic BP, mm Hg				
Age 36, seated	286	—	78 (10)	79 (72 to 84)
Age 43, seated	303	—	80 (9)	79 (74 to 85)
Age 53, seated	310	—	83 (12)	82 (76 to 89)
Age 60–64, seated	317	—	76 (10)	76 (70 to 82)
Age 69, seated	315	—	73 (10)	73 (66 to 80)
Age 70, lying	315	—	73 (10)	73 (66 to 80)
Age 70, standing	315	—	80 (11)	80 (72 to 87)
FHS-CVS				
Age 36	286	—	2.9 (1.8)	2.7 (1.5 to 3.5)
Age 53	307	—	11.7 (7.1)	10.6 (6.3 to 15.6)
Age 69	312	—	25.3 (13.2)	23.3 (14.5 to 34.3)
Follow-up (phase 2) plasma NfL, pg/mL	312	—	16.7 (7.9)	15 (11.8 to 19.4)

Abbreviations: Aβ = β-amyloid; CL = Centiloid; DBP = diastolic blood pressure; FHS-CVS = Framingham Heart Study Cardiovascular Risk Score; HAR = hippocampal atrophy rate; IQR = interquartile range; NfL = neurofilament light chain; SBP = systolic blood pressure; WMHV = white matter hyperintensity volume.

### Relationship Between Baseline (Phase 1) WMHV and Rate of WMHV Change

**Figure 3:**
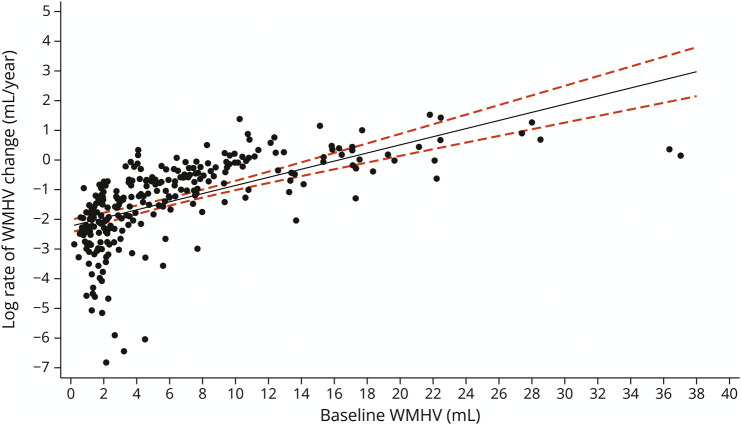
Regression Margin Plot Showing the Relationship Between Baseline WMHV and Log WMHVR Regarding relationship between baseline WMHV and subsequent WMHVR over 2.4 years (log transformed), the plot shows predicted marginal mean log WMHVR and 95% CI from the linear regression model adjusted for TIV, age at baseline, and sex. TIV = total intracranial volume; WMHV = white matter hyperintensity volume; WMHVR = WMHV change rate.

Because the WMHVR data were log transformed when used as a dependent variable (in this model only), there was a reduction in sample size (N = 266) due to the removal of negative values (indicating decrease in WMHV from baseline to follow-up). For every additional 1 mL WMHV at baseline, there was an associated increase (β = 0.14, 95% CI 0.11–0.17) in log WMHVR over the following 2.4 years when adjusting for TIV, age at baseline, and sex (*p* < 0.001) (Figure 3).

### Relationship Between Rate of WMHV Change and Rate of Hippocampal Atrophy

**Figure 4:**
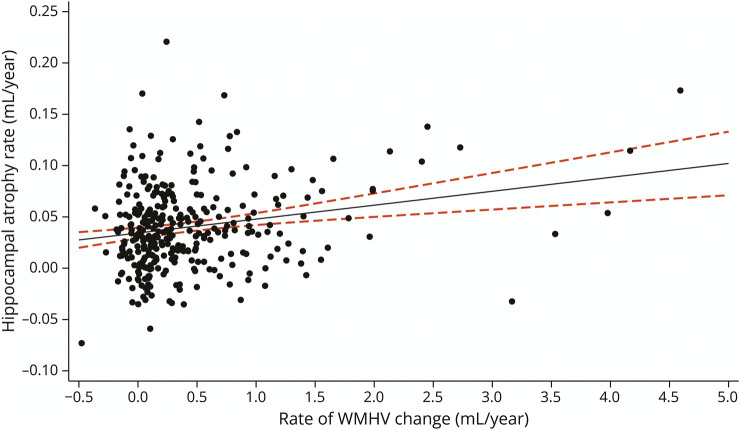
Regression Margin Plot Showing the Relationship Between WMHV Change Rate and Hippocampal Atrophy Rate Regarding relationship between rate of WMHV change and hippocampal atrophy rate over 2.4 years, the plot shows predicted marginal mean hippocampal atrophy rate and 95% CI from the linear regression model adjusted for TIV, age at baseline, and sex. TIV = total intracranial volume; WMHV = white matter hyperintensity volume.

The mean total hippocampal volume in this sample was 6.20 mL, with a mean HAR of 0.04 mL/y (N = 317). For every 1 mL/y increase in WMHVR, there was an associated 0.014 mL/y (95% CI 0.005–0.022) increase in HAR (*p* = 0.002), adjusting for TIV, age at baseline, and sex (Figure 4). There was an attenuated but significant effect of WMHVR on HAR when further adjusting for whole-brain atrophy, where each 1 mL/y increase was associated with 0.008 mL/y (95% CI 0.003–0.013) HAR (*p* = 0.004).

### Influence of Life-Course Cardiovascular Risk Factors

The association between higher WMHVR and increased HAR was not markedly attenuated with additional adjustment for seated SBP or DBP measurements at ages 36, 43, 53, 60–64, and 69 as well as lying and standing SBP or DBP at 70 years in separate models (eFigure 5, models 2.1.1–2.7.2, *p* ≤ 0.005, all models). Similarly, adjustment for FHS-CVSs at ages 36, 53, and 69 did not attenuate this relationship (eFigure 5, models 3.1–3.3, *p* ≤ 0.003, all models).

### Influence of AD Pathology and Genetic Risk

**Figure 5:**
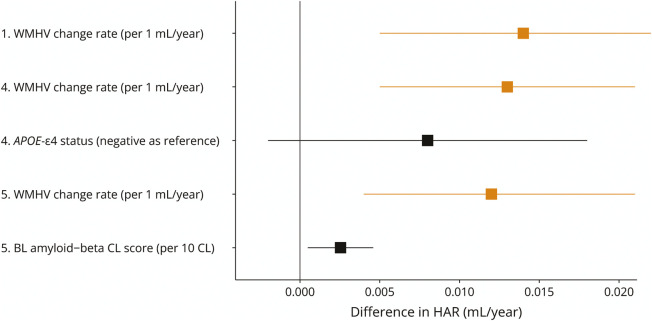
Forest Plot Showing the Effects of WMHVR on Hippocampal Atrophy Rate, While Accounting for the Effects of APOE-ε4 Status and Aβ in Separate Models Forest plot showing coefficients and 95% CIs for the influence of WMHVR and additional neurologic variables of interest in separate models on HAR as the outcome. The coefficient reflects the difference in hippocampal atrophy rate (mL/y) associated with levels of independent variables stated in brackets. All models were adjusted for sex, age at baseline, and TIV. Dashed horizontal lines separate the different models. The sample size for each model is determined by the N for each additional independent variable given in Table 1. BL = baseline assessment; CL = Centiloid; HAR = hippocampal atrophy rate; NfL = neurofilament light chain; TIV = total intracranial volume; WMHV = white matter hyperintensity volume; WMHVR = WMHV change rate.

To examine the influence of *APOE*-ε4 status and phase 1 Aβ CL score on the association between WMHVR and HAR, we further accounted for these measurements in 2 separate linear regression models (Figure 5, models 4 and 5). Neither markedly attenuated the association between higher WMHVR and increased HAR (*p* ≤ 0.005, both models), but higher baseline Aβ levels were independently associated with increased HAR (*p* = 0.015) after adjusting for age, sex, TIV, and WMHVR. In a separate regression model including an interaction term between WMHVR and Aβ, there was no evidence of a significant interactive effect between WMHVR and baseline Aβ CL score (*p* = 0.63).

### Combined Model

When including all independent variables and covariates (Table 1, models 1–5) in a single, combined linear regression model (eFigure 6, N = 257), there was an slightly attenuated but significant effect with each 1 mL/y increase in WMHVR associated with a higher HAR of 0.013 mL/y (95% CI 0.004–0.022) (*p* = 0.005).

### Complete Case Analysis

Models 1–5 (Table 1) were repeated with the inclusion of only individuals with complete data (measurements for all variables) to account for the possibility that the differences in the influence of WMHVR on HAR while accounting for additional variables was due to reductions in sample size, rather than the influence of the additional variable. When analyzing only individuals who had data for all observations (N = 257), there were no meaningful changes to the influence of higher WMHVR on increased HAR in any model in comparison with the main sample (eFigures 7 and 8).

### Sensitivity Analysis

Including individuals with all major neurologic disorders other than cortical stroke or MS (N = 330) slightly increased the effect of higher WMHVR on increased HAR so that a 1 mL/y higher WMHVR was associated with a higher HAR of 0.016 mL/y (95% CI 0.007–0.025) (eFigures 9 and 10, *p* = 0.001), adjusting for sex, age, and TIV.

### Variance in HAR Uniquely Explained by WMHVR

Calculated using semipartial *R*^2^, the percentage of variance in HAR uniquely explained by WMHVR was 4.6% when adjusting for sex, age, TIV, and scan interval. This remained ≥3.8% when further adjusting for all BP and FHS-CVS measurements as well as Aβ and *APOE*-ε4 status in separate models and was 3.6% when adjusting for all variables in a fully adjusted model. The total effect on HAR explained by all independent variables in the model (*R*^2^) was 29.3% when adjusting for all variables in the same model (eTable 2).

### Indirect Effects of WMHVR on HAR With Phase 2 Plasma NfL

In a post hoc analysis, we hypothesized that axonal degradation may provide a mechanistic explanation for the relationship between WMHVR and HAR (with WMHVR reflecting increases in WM damage, and this WM damage leading to reduced stimulus to the hippocampus, causing it to atrophy at a greater rate). Using structural equation modeling, we tested whether follow-up (phase 2) plasma NfL (a marker of axonal degradation) mediated the relationship between WMHVR and HAR. From this investigation, there was no statistically significant indirect effect of WMHVR on HAR mediated by phase 2 plasma NfL (*p* = 0.4).

## Discussion

While previous research in this cohort has shown that greater WMH accumulation by age 70 is related to higher HAR,^11^ this study aimed to determine whether increases in WMHV after age 70 were also related to higher HAR over the same period of approximately 2.5 years. The findings derived from this population-based study of aging in individuals of almost identical age support our hypothesis that WMHVR is related to HAR, in a process that is additive to but independent of the contribution of *APOE*-ε4 status and Aβ burden. However, the dependence on CVRF was less than expected. These findings show that continual increases in WMHV, and not just total accumulation by age 70, are related to increased HAR after 70, although those with higher baseline WMHV are likely to have higher WMHVR. Although the effects of WMHVR on HAR were relatively modest in this mostly healthy, normotensive sample, they were greater than the effects of baseline Aβ and may be higher in individuals with higher WMHVR, mild cognitive impairment (MCI), or AD (supported by the greater effects observed in the sensitivity analysis including individuals with neurologic disorders).

When adjusting for whole-brain atrophy, the effect of WMHVR on HAR remained significant, meaning that this effect was not explained by the influence of WMHVR on generalized brain atrophy as a whole, rather that WMHVR disproportionately affects the hippocampus. This suggests that the hippocampus is particularly vulnerable to presumably systemic processes that also lead to WMH progression, such as cSVD-related hypoxia, inflammation, or increased blood-brain barrier permeability. The latter 2 may also lead to impaired Aβ and p-tau clearance, which are known to have an effect on hippocampal atrophy.

The rates of WMHV change in this sample are consistent with other studies and, as has been shown previously, the greater the burden at baseline, the greater the increase in burden over time.^31^ Previous analyses in this cohort have shown that high and increasing BP from midlife is associated with higher WMHV around age 70. Relationships between poorer cardiovascular health and hippocampal atrophy in individuals with cognitive impairment have also been demonstrated.^32^ Based on these previous findings, we hypothesized that the association between WMHVR and HAR would be somewhat dependent on CVRF, particularly hypertension in midlife. However, accounting for CVRF at different stages of adulthood did not meaningfully attenuate the association between increased WMHVR and HAR. This suggests that poorer cardiovascular health from midlife onward is associated with higher WMHV measured at age 70, but once WMHs are established, they continue to expand in association with hippocampal atrophy, independent of cardiovascular disease–related processes that occurred between ages 36 and 70 in this sample. However, a study containing more hypertensive individuals than this mostly normotensive sample may yield different results.

Multiple studies have sought to determine whether WMH accumulation is directly related to Aβ pathology, with varying results.^33,34^ Our findings here are supportive of an additive but independent effect of global WMHV increases and Aβ deposition on hippocampal atrophy, which is consistent with previous cross-sectional findings in this cohort^11^ and others.^12^

Although *APOE*-ε4 status confers an elevated risk of AD development and has been associated with hippocampal atrophy in individuals with MCI and AD,^35^ there was not a significant relationship between *APOE*-ε4 status and HAR in our main analysis sample, nor did it account for the association between WMHVR and HAR. This is likely explained by the absence of neurologic diseases in the data set, or that *APOE*-ε4 carriers only represent 30% of the sample, resulting in a reduction in statistical power.

This study did not elucidate the processes behind the relationship between this post-70 increase in WMHVR and associated HAR. We hypothesized that axonal degradation measured with follow-up plasma NfL may mediate the relationship between WMHVR and HAR, with the rationale being that WM damage reflected by WMHs and measured directly with NfL would lead to reduced connectivity and stimulus to the hippocampus, causing it to atrophy at a faster rate. NfL did not mediate this association, so it is likely instead that systemic processes, potentially increased blood-brain barrier permeability, and inflammation secondary to sustained cSVD, will explain this association. Because a modest amount (4.7%) of HAR was uniquely explained by WMHVR and only 29.3% was explained by all covariates, CVRF, *APOE*-ε4 status, and Aβ levels combined, most of HAR is left unexplained. Therefore, investigations into the effects of inflammation, as well as p-tau and TDP-43 (both known to influence hippocampal volume^36,37^), which were, as yet, unavailable in this sample, will be informative.

It is worth noting that the results suggesting that the WMHVR-HAR relationship was independent of CVRF and Aβ may in part be due to the use of only a global measure of WMHV change. Studies have shown differing spatial distributions of WMHs associated with different pathologic causes, specifically frontal WMH accumulation arising from vascular processes and posterior WMH accumulation due to Aβ accumulation.^38^ Analyzing the spatial distribution of WMH changes may provide more insights into the mechanisms behind this association that were not captured when measuring WMH changes globally.

The main strengths of this study were that participants were almost identical in age and the geographic distribution of study members is similar to that of the British population in general. In addition, data were collected at a single center using a standardized protocol and a single PET/MRI scanner, and our automated method of WMH segmentation (BaMoS) has been shown to have high agreement with semiautomated segmentation methods and is robust to differing WMH loads.^39^

There are, however, limitations to this study. All participants in this study, who were recruited to the NSHD from birth in 1946, were White and were born in Great Britain. In addition, the demanding nature of the testing conducted at UCL for this study causes a selection bias for participants with better physical health and cognitive ability. Participants in this substudy (Insight46) had slightly higher socioeconomic status and educational attainment than the NSHD cohort as a whole. Naturally, those who died before Insight46 could not be included, which results in retention bias. Thereby, people from backgrounds other than White British, those with lower income and educational attainment, and those with poorer health are under-represented. This means the findings of this study (particularly related to cardiovascular risk, which is known to vary by ethnic group, socioeconomic status, and physical health^40,41^) cannot yet be generalized to other areas of the world nor the ethnically diverse population of Great Britain today without being reproduced in more diverse cohorts.

In addition, WMHV in this sample of healthy individuals is relatively low in comparison with MCI and AD, and we excluded individuals from this study who had evidence of cortical stroke or autoimmune demyelination and included few individuals with other neurologic disorders only in sensitivity analysis. Although the rationale for these exclusion criteria is valid to prevent stroke or MS lesions being mischaracterized as WMH, individuals who have suffered stroke are likely to also have higher WMH burden, thus excluding the more extreme cases of cSVD. Future work is needed to classify and include these individuals; however, this was beyond the scope of this study. Furthermore, the time interval between MRI measurements (∼2.5 years) was relatively short and so the continued effects of WMHVR on HAR will only be determined once additional time points are available from the next phase of this study, or if they can be replicated in other longitudinal studies with longer follow-up periods.

The linear regression model investigating effects of WMHVR on HAR was adjusted for whole-brain BSI to determine whether WMHVR disproportionately affected the hippocampus relative to the whole brain. Using whole-brain BSI in analysis with WMHVR has a methodological limitation in that periventricular WMHs seem hypointense on T1 (the sequence used for BSI). Therefore, the expansion of these WM T1 hypointensities contiguous with the CSF in the lateral ventricles may be conflated with ventricular expansion measured with BSI, overestimating atrophy, which could induce an association between WMHVR and ventricular expansion (which contributes to whole-brain BSI). Further work is required to account for this potential measurement bias and determine whether reduction or elimination of this bias influences the relationship between WMHVR and HAR when adjusting for whole-brain BSI in linear regression models.

Increases in WMHV are related to disproportionate hippocampal atrophy over the same period in the Insight46 substudy of the 1946 British Birth Cohort. While AD-specific pathology is often assumed to be the main driver of accelerated hippocampal atrophy, we show that HAR is also related to progressing WM disease, showing the relevance of continued WMHV accumulation over time. This has implications in understanding the additive effects of presumed cerebrovascular disease and Aβ on brain aging and in using neuroimaging biomarkers to assist with diagnosis and monitoring of early AD. Determining the mechanisms behind this relationship will aid the understanding of brain aging and dementia.

## Glossary

Aβ: β-amyloid
AD: Alzheimer disease
BaMoS: Bayesian Model Selection
BP: blood pressure
BSI: boundary shift integral
CL: Centiloid
cSVD: cerebral small vessel disease
CVRF: cardiovascular risk factor
DBP: diastolic BP
FHS-CVS: Framingham Heart Study Cardiovascular Risk Score
FLAIR: fluid-attenuated inversion recovery
HAR: hippocampal atrophy rate
KN: k-means–normalized
MCI: mild cognitive impairment
MS: multiple sclerosis
NfL: neurofilament light chain
NSHD: National Survey of Health and Development
p-tau: phosphorylated tau
SBP: systolic BP
TIV: total intracranial volume
UCL: University College London
WMH: white matter hyperintensity
WMHV: WMH volume
WMHVR: WMHV change rate
